# Biofunctionalization of decellularized porcine aortic valve with OPG-loaded PCL nanoparticles for anti-calcification

**DOI:** 10.1039/c9ra00408d

**Published:** 2019-04-16

**Authors:** Yang Li, Yu Zhang, Jing-Li Ding, Ji-Chun Liu, Jian-Jun Xu, Yan-Hua Tang, Ying-Ping Yi, Wei-Chang Xu, Wen-Peng Yu, Chao Lu, Wei Yang, Jue-Sheng Yang, Yi Gong, Jian-Liang Zhou

**Affiliations:** Department of Cardiovascular Surgery, The Second Affiliated Hospital of Nanchang University No. 1, Mingde Road Nanchang 330000 China zhoujianliang2010@163.com +86 13767117511; Department of Cardiovascular Surgery, Renji Hospital, School of Medicine, Shanghai Jiaotong University Shanghai China; Department of Gastroenterology, The Second Affiliated Hospital of Nanchang University Nanchang China; Department of Science and Education, The Second Affiliated Hospital of Nanchang University Nanchang China

## Abstract

Decellularized valve stents are widely used in tissue-engineered heart valves because they maintain the morphological structure of natural valves, have good histocompatibility and low immunogenicity. However, the surface of the cell valve loses the original endothelial cell coverage, exposing collagen and causing calcification and decay of the valve in advance. In this study, poly ε-caprolactone (PCL) nanoparticles loaded with osteoprotegerin (OPG) were bridged to a decellularized valve using a nanoparticle drug delivery system and tissue engineering technology to construct a new anti-calcification composite valve with sustained release function. The PCL nanoparticles loaded with OPG were prepared *via* an emulsion solvent evaporation method, which had a particle size of 133 nm and zeta potential of −27.8 mV. Transmission electron microscopy demonstrated that the prepared nanoparticles were round in shape, regular in size, and uniformly distributed, with an encapsulation efficiency of 75%, slow release *in vitro*, no burst release, no cytotoxicity to BMSCs, and contained OPG nanoparticles *in vitro*. There was a delay in the differentiation of BMSCs into osteoblasts. The decellularized valve modified by nanoparticles remained intact and its collagen fibers were continuous. After 8 weeks of subcutaneous implantation in rats, the morphological structure of the valve was almost complete, and the composite valve showed anti-calcification ability to a certain extent.

## Abstract 2

A novel composite valve with controlled release OPG was prepared by introducing tissue engineering technology and nano drug-loading system to introduce anti-calcification biological factor OPG on the decellularized valve.

## Graphical abstract

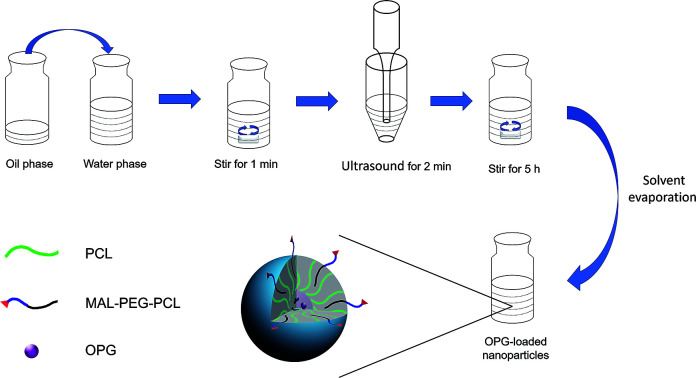

## Introduction

Heart valve disease is the leading cause of cardiovascular death, where calcified aortic valve disease (CAVD) remains dominant.^1^ The incidence of CAVD has increased continuously with the increase in the aging population, which ranks second among the cardiovascular diseases.^2^ To date, there is no effective drug for the prevention or treatment of CAVD, and the main clinical treatment for CAVD is valve replacement surgery. However, the existing mechanical valves require the long-term use of anticoagulant drugs. The same or different types of biological valves are calcified and have a high chance of decay, which affect the quality of life of patients after surgery, resulting in great suffering.^3^ Therefore, the development of new types of anti-calcification heart valves is necessary, which remains the goal of clinical researchers.

Tissue-engineered heart valves (TEHV) are an ideal valve replacement approach that overcomes the deficiencies of existing mechanical and biological valves. They have broad application prospects clinically due to their characteristics of self-renewability and remodeling, no anticoagulation, low immunogenicity, durability, calcification, *etc.*^4,5^ The scaffold materials used for TEHV mainly include polymer materials and natural decellularized materials. Decellularized valve stents are widely used in TEHV because they maintain the morphological structure of natural valves, have good histocompatibility and low immunogenicity.^6,7^ However, the surface of the cell valve loses its original endothelial cell coverage, exposing collagen and causing calcification and decay of the valve in advance.^8^ Therefore, it is of great value to biomodify decellularized valves for anti-calcification.

Nano drug-loading systems are a new type of drug carrier with broad application prospects for development. Nanoparticle drug-loading systems enhance the stability of protein drugs and have excellent sustained-release and controlled-release properties, helping to achieve local therapeutic effects.^9^ The drug-loading materials mainly include natural and synthetic polymers. Among them, nanoparticles prepared using synthetic polymer materials have a large range of applications due to their high bioavailability, good encapsulation, control release and non-toxic properties.^10,11^ Polycaprolactone (PCL) is a hydrophobic biodegradable polymer with good biocompatibility due to its linear aliphatic polyester, which is obtained *via* ring-opening polymerization using ε-caprolactone (ε-CL). However, PCL degrades rather slowly and has low biocompatibility with soft tissues, which restrict its clinical application.^10^ Polyethylene glycol (PEG) is a long-chain polymer with high hydrophilicity, non-toxicity and good histocompatibility. It has a wide range of applications in the medical and health fields.^12^ The PEG–PCL copolymer is formed by binding PEG to PCL, which has greatly improved hydrophilicity, biodegradability and mechanical properties. Therefore, PEGylated PCL nanoparticles are considered to be more suitable for drug delivery than PCL nanoparticles alone.^13^

Osteoprotegerin (OPG) is a soluble secretory glycoprotein belonging to the TNF receptor superfamily. Its N-terminal cysteine is required for the formation of disulfide bonds in homodimers.^14^ OPG competitively prevents RANK ligands from binding to the nuclear factor κB receptor activating factor and inhibits the abnormal osteogenesis of vascular and valvular stromal cells. Thus, the relative or absolute deficiency of its secretion may be an important cause for valvular calcification.^15^

Previous studies have shown that PEG cross-linked to decellularized heart valves improved the mechanical and biological properties of the valve stent.^16^ In this study, the water-soluble protein OPG was encapsulated in MAL–PEG–PCL-modified PCL nanoparticles *via* a nano drug-loading system, which improved the bioavailability of OPG, and then the nanoparticles were terminated by Michael addition reaction. The combination of unsaturated carbon–carbon double bonds and thiolated decellularized valves allowed the nanoparticles to bridge the decellularized valve for the construction of a novel composite valve with sustained release function.

## Materials and methods

### Materials

The materials used included polycaprolactone (PCL, *M*_n_ 10 000 Da, Sigma-Aldrich, U.S), maleimide–poly(ethylene glycol)–poly(ε-caprolactone) (MAL–PEG–PCL, *M*_n_ 5000 Da, Xi'an Ruixi Biotech, China), Dulbecco's Modified Eagle Medium (DMEM, Hyclone), fetal bovine serum (FBS, Gibco, Australia), osteoprotegerin (OPG, PeproTech, U.S.), OPG ELISA kit (Cloud-Clone Corp, U.S), soybean phosphatidylcholine (SPC, Shanghai Taiwei Biotech, China), Cell Counting Kit 8 (CCK-8, Beijing Transgen Biotech, China), dexamethasone, ascorbic acid, β-glycerol phosphate (Sigma-Aldrich, U.S.), QuantiChrom™ Calcium Assay Kit (BioAssay Systems, U.S), Hematoxylin and Eosin staining kit (H&E staining, Wuhan Boster Biological Technology, China), Masson's Trichrome Stain Kit (MASSON Staining, Genmed Scientifics. Inc, U.S), and osteocalcin antibody (OCN, Wuhan Boster Biological Technology, China). Male Sprague-Dawley (SD) rats weighing 175 ± 26 g, 4 weeks old, were provided by the Experimental Animal Center of Nanchang University. All research protocols were approved by the Institutional Animal Care and Use Committee of the Second Affiliated Hospital of Nanchang University.

### Methods

#### Preparation of nanoparticles loaded with OPG

**Fig. 1:**
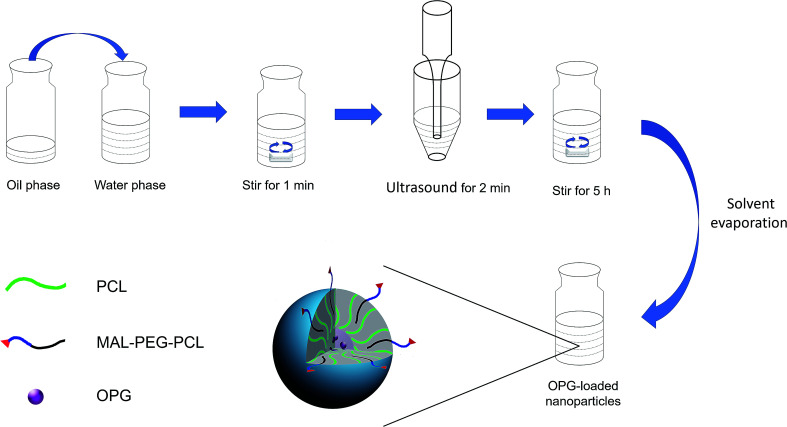
Preparation of OPG-loaded nanoparticles *via* the emulsification solvent evaporation method.

MAL–PEG–PCL-modified PCL nanoparticles were prepared *via* an emulsion solvent evaporation method. The schematic diagram of the preparation of nanoparticles loaded with OPG is shown in Fig. 1. Briefly, OPG (1 μg mL^−1^) was dissolved in sterile trihydrated water and the concentration was adjusted to 1 μg mL^−1^. Then 10 mg soybean phospholipid was accurately weighed and dissolved in 1 mL of *t*-butanol, and then in 1 mL of 1 μg mL^−1^ OPG aqueous solution was added to 1 mL of 10 mg mL^−1^ soybean phospholipid/*tert*-butanol. The solution was thoroughly blown with a pipette, pre-frozen at −60 °C for 2 h in a freeze dryer, and then dried for 22 h to obtain an OPG–phospholipid complex. The blank phospholipid complex was prepared as described before. As shown in Fig. 1, the O/W emulsion was first prepared, and then 4 mg of MAL–PEG–PCL, 12 mg of PCL and 10 mg of OPG–phospholipid complex were dissolved in 1.5 mL of methylene chloride as the oil phase (O), and 6 mL 2% (w/v) PVA aqueous solution as the aqueous phase (W). The oil phase was slowly added to the aqueous phase and magnetically stirred for 1 min. The stirred mixed solution was ultrasonicated in an ice bath (SCIENTZ-II D, Ningbo Xinzhi Bio Technology Co., Ltd.), ultrasonicated at a power of 60 W for 2 min, and then stirred at room temperature for 5 h to fully volatilize the methylene chloride and finally obtain the MAL–PEG–PCL-modified OPG nanoparticles (OPG-NPs). Non-loaded OPG nanoparticles (NL-NPs) were prepared similarly, except that a blank phospholipid complex was added.

#### Characterization of nanoparticles loaded with OPG

##### Particle size and zeta potential

The particle size and zeta potential distribution of the nanoparticles were measured *via* laser light scattering (LLS, Zetasizer Nano, Malvern, UK). 1 mL of the prepared nanoparticle suspension was diluted 4 times with triply distilled water, thoroughly mixed, and then the particle size and zeta potential distribution were determined using a measuring instrument.

##### Transmission electron microscopy

The morphology and particle diameter of the nanoparticles were observed *via* transmission electron microscopy (TEM, JEM-2100, JEOL Ltd.). The prepared nanoparticle suspension was diluted 100 times with triply distilled water, and thoroughly mixed. Then 10 μL of the diluted nanoparticle suspension was dropped on a copper mesh covered with a support film, which was placed in a ventilated area to dry naturally and then dropped in 2% phosphorus. The tungstic acid solution was dried for 2 min. After drying, the copper mesh was placed under TEM to observe the morphology of the nanoparticles.

##### Encapsulation efficiency

The content of encapsulated OPG in the nanoparticles was detected using an enzyme-linked immunosorbent assay (ELISA). Briefly, the prepared nanoparticle suspension was diluted to 10 mL with PBS and mixed thoroughly. Subsequently, 6 mL of nanoparticle suspension was aspirated by ultracentrifugation (32 000 rpm, 20 min, 4 °C), and then the supernatant was collected. 1 mL of nanoparticle suspension was added to an EP tube and 20 μL of dimethyl sulfoxide (DMSO) was added, and then the mixture was thoroughly mixed.

According to the manufacturer's instructions for the OPG ELISA kit, 100 μL of standard, supernatant and DMSO and nanoparticle suspension were added to the corresponding wells of the plate, incubated at 37 °C for 1 h, and then the liquid in the well was discarded. Subsequently, 100 μL of test solution A was added to each well, incubated at 37 °C for 1 h, and then the liquid in the well discarded. 350 μL washing solution was added to each well, which was washed 3 times. Then the enzyme plate was dried and 100 μL of test solution B added to each well and incubated at 37 °C for 30 min. The liquid in the well was discarded and the well was washed with washing solution 5 times, and then 90 μL of TMB substrate solution was added to each well, and color development was observed at 37 °C for 20 min in the dark. Then 50 μL of reaction stop solution was added to each well, and finally the ELISA plate was placed on the multi-functional enzyme. The absorbance of each well was measured at 450 nm by placing it on a standard instrument (VARIOSKAN, Thermo Fisher Scientific, USA). The supernatant OPG content (*M*_0_) and the total OPG content of the nanoparticle suspension (*M*_1_) were calculated separately. The nanoparticle encapsulation efficiency (EE) was calculated as follows: EE (%) = (*M*_1_ − *M*_0_)/*M*_1_ × 100%.

##### OPG cumulative release of nanoparticles

PBS (pH 7.4) was used as the release medium for the release of the nanoparticles *in vitro*, and the released OPG content was detected *via* ELISA. The prepared nanoparticle suspension was diluted to 10 mL with PBS and mixed thoroughly. 6 mL of nanoparticle suspension was placed in an ultracentrifuge tube and centrifuged at 32 000 rpm for 20 min at 4 °C, the supernatant discarded, and the nanoparticles resuspended in 5 mL PBS. The pellet was placed in a constant temperature water bath oscillator (SHA-BA, Changzhou Langyue Instrument Manufacturing Co., Ltd.) and continuously shaken at 37 °C and 60 rpm. After intervals of 6 h, 12 h, 24 h, 2 d, 3 d, 5 d, and 7 d, the tubes were taken out of the shaker bath, centrifuged at 4 °C and 32 000 rpm for 20 min, and the supernatant collected. The nanoparticle pellet was then resuspended in 5 mL PBS, placed in a constant temperature water bath shaker, and then the supernatant was collected again at the next time point.

##### Isolation and culture of rat bone marrow mesenchymal stem cells (rBMSCs)

According to the method described by Li *et al.*,^17^ 3 week-old male Sprague-Dawley rats were sacrificed by cervical dislocation. The rats were immersed in 75% ethanol for 15 min. The femur and tibia of the rats were isolated under aseptic conditions. The bone marrow cavity was washed with a medium containing 10% FBS, 100 U mL^−1^ penicillin, and 100 μg mL^−1^ streptomycin using a 5 mL syringe. The cell suspension was centrifuged at 1000 rpm for 5 min, the supernatant was discarded, the cells were re-suspended in complete medium, thoroughly mixed and then placed in a new culture flask at 37 °C in a 5% CO_2_ incubator (HERAcell 150i, American Thermo Scientific).

##### Flow cytometry

Second generation BMSCs were collected, trypsinized and then centrifuged (1000 rpm, 5 min). The supernatant was discarded, the cells were suspended in sterile PBS and the cell density was adjusted to 1 × 10^6^/mL, and 500 μL of cell suspension was pipetted into a flow tube. 10 μL of FITC-labeled CD29, CD90, and CD45 and 10 μL of PE-labeled CD34 antibodies were added to each flow tube. The isotype IgG antibody was used as an isotype control, incubated at 4 °C for 30 min in the dark, and then placed in a flow cytometer (FACS Calibur, USA, BD) to detect the expression of each antibody.

##### Cytotoxicity of nanoparticles

The effect of the OPG nanoparticles on the proliferation of BMSCs was detected *via* the CCK-8 method. The prepared nanoparticle suspension was centrifuged at a low temperature (32 000 rpm, 20 min, 4 °C), the supernatant was discarded, resuspended in 2 mL of sterile PBS, and then filtered through a 0.22 μm sterile membrane for use. The experiment involved the blank nanoparticle group (NL-NPs), OPG-loaded nanoparticle group (OPG-NPs), PBS and blank control group. Third generation BMSCs were inoculated on a 96-well culture plate at a density of 1 × 10^4^/mL, each well was inoculated with 100 μL of cell suspension, placed at 37 °C, incubated in a 5% CO_2_ incubator for 24 h, and then the corresponding 10 μL NL-NPs, OPG-NPs, and PBS were added to the wells. Only 100 μL PBS was added to the blank wells. After 12 h, 24 h, and 48 h, the cell culture was replaced with new culture medium, followed by the addition of 10 μL CCK-8 test solution. The cell culture was left to incubate for 2 h and then placed on an enzyme label instrument. The absorbance values of the respective wells were measured at 450 nm on a detector.

##### Osteogenic differentiation

Complete medium for osteogenic differentiation containing DMEM high glucose medium, 10% FBS, 100 U mL^−1^ penicillin, 100 μg mL^−1^ streptomycin, 10 mmol L^−1^ β-glycerophosphate, 50 μg mL^−1^ ascorbic acid, and 100 nM dexamethasone, hereinafter referred to as DAG, induces the differentiation of BMSCs into osteoblasts.^17^ An appropriate amount of prepared OPG nanoparticle (OPG-NPs) and blank nanoparticle (NL-NPs) suspension was taken, centrifuged at low temperature (32 000 rpm, 20 min, 4 °C), the supernatant discarded, and then re-suspended in 2 mL sterile PBS. Subsequently, it was filtered through a 0.22 μm sterile filter for further use. The third generation BMSCs were seeded in a 24-well culture plate at a density of 2 × 10^4^/mL, and cultured at 37 °C in a 5% CO_2_ incubator. The DAG, OPG-NPs, and NL-NPs groups included 40 μL PBS, OPG-NPs, NL-NPs and 500 μL DAG mixed solution and 540 μL complete culture only was added in the normal control group. Six replicate wells for each experimental group were prepared. When the cell fusion reached 60–70%, the medium in the wells of the culture plate was carefully discarded, thoroughly rinsed with sterile PBS, and then the respective mixture was added to each well. The medium was changed once a week. Calcification was evaluated on days 7, 14, and 21 after osteogenic differentiation.

##### Alizarin red staining

Alizarin red forms a red complex with calcium salts, and the formation of calcium nodules was observed by alizarin red staining. At day 21, the plate was washed with PBS, 500 μL of 4% paraformaldehyde was added to each well, fixed at room temperature for 30 min, and rinsed thoroughly with PBS. Subsequently, 500 μL of alizarin red dye solution was added to each well, stained for 5 min, rinsed with PBS and then observed under a light microscope.

##### Calcium deposition test

The calcium deposition in each group was quantitatively detected using the calcium ion content in the calcium nodules. Firstly, 500 μL of 0.6 N HCL was added to each well, and then the mixture was incubated overnight at 37 °C. The extracts of each group were collected the next day, centrifuged at 2500 rpm for 5 min, and then the supernatant was collected. According to the manufacturer's instructions of the calcium quantitative test kit, 5 μL of supernatant was added to the bottom of a 96-well culture plate, and then 200 μL of the test solution was added to each well. After standing at room temperature for 5 min, the absorbance values were measured at 630 nm.

##### Preparation of decellularized valve modified with OPG nanoparticles

Fresh porcine hearts were obtained from the local slaughterhouse under clean conditions, and then the porcine aortic valve (PAV) was cut in a sterile environment. The aortic valve was then preserved in PBS containing antibiotics (0.1 mg mL^−1^ streptomycin, 100 U mL^−1^ penicillin, and 0.25 μg mL^−1^ amphotericin B). The obtained aortic valve was placed in PBS containing 0.05% (w/v) trypsin and 0.02% (w/v) EDTA at 37 °C for 12 h. It was further placed in PBS containing 1% (v/v) Triton X-100 at 4 °C for 48 h, washed with PBS, and then treated with PBS containing nuclease (DNase I 0.2 mg mL^−1^ and RNase A 20 μg mL^−1^) at 37 °C for 1 h. After depletion with PBS, a decellularized porcine aortic valve (DPAV) was obtained. According to Chen,^18^ the prepared decellularized valve was reacted with thiolation reagent SATA at 37 °C for 2 h, and then the reaction was terminated by elution with PBS. Subsequently, it was reacted with hydroxylamine hydrochloride at 37 °C for 2 h to protect the acetylated thiol group, and washed with PBS for thiolation.

The thiolated DPAV was immersed in a diluted OPG-NPs solution, and then placed on a constant temperature shaker at 37 °C, 60 rpm in the dark for 8 h. The nanoparticles that were not bound to the valve were washed away with PBS to finally obtain the OPG-loaded PCL nanoparticle-modified composite valve (OPG-NPs–DPAV). The nanoparticle-modified DPAV without OPG was also prepared using this method (NL-NPs–DPAV).

#### Characterization of the complexed DPAV

##### HE and MASSON staining

The valves in each group (PAV, DPAV, OPG-NPs–DPAV, and NL-NPs–DPAV) were fixed with 4% paraformaldehyde for 24 h, embedded in paraffin, sectioned, and stained with reference to HE and MASSON staining kit instructions. The decellularization effect and the valvular collagen fibers were then observed under a light microscope.

##### Scanning electron microscopy

DPAV and OPG-NPs–DPAV were fixed in 2.5% glutaraldehyde for 24 h, dehydrated using an ethanol gradient, dried at the CO_2_ critical point, sprayed with gold by ion sputtering, and then placed under a scanning electron microscope (FEI Quanta 200F, American EI) to observe the combination of granules and decellularized valves.

##### Attenuated total reflection Fourier infrared spectroscopy (ATR-FITR)

OPG-NPs–DPAV, NL-NPs–DPAV and DPAV were plated on a watch glass, frozen in a freeze dryer at −56 °C for 3 h, and then vacuum dried for 20 h. The dried groups of the valves were ground into a powder and thoroughly mixed with potassium bromide. An appropriate amount of each sample was utilized to form a tablet, and then its infrared absorption spectrum was measured by Fourier transform infrared spectroscopy (FTIR, Nicolet 5700, Nico, USA).

##### Anti-calcification of composite valve *in vivo*

The rat subcutaneous implantation model is used as a routine method for the detection of the anti-calcification biological properties of biological valves.^19^ To simulate the calcification of valves in each group (PAV, DPAV, OPG-NPs–DPAV, and NL-NPs–DPAV) *in vivo*, 4 week-old male SD rats were divided into 4 groups, with 9 in each group, and then anesthetized by intraperitoneal injection of 1% sodium pentobarbital. The subcutaneous tissue was blunt free. The valves of each group (1 cm × 1 cm) were sutured and fixed on the fascia, with one valve in each incision. Two valves were placed per rat. The back incision was sutured and then the back suture was removed after 7 days. The animals were sacrificed by CO_2_ asphyxiation after 2, 4 and 8 weeks and the samples were retrieved. After fixing in 4% paraformaldehyde, the samples were embedded in paraffin and then sliced. HE staining and osteocalcin (OCN) immunohistochemical staining were performed. The infiltration of valve cells, collagen fiber shape and OCN expression were observed under a light microscope.

##### Statistical analysis

Data are expressed as mean ± standard deviation. Statistical differences in the measured properties between groups were determined using one-way ANOVA with Student–Newman–Keuls. *P* values < 0.05 were considered to be statistically significant.

## Results

### Characterization of OPG nanoparticles

**Fig. 2:**
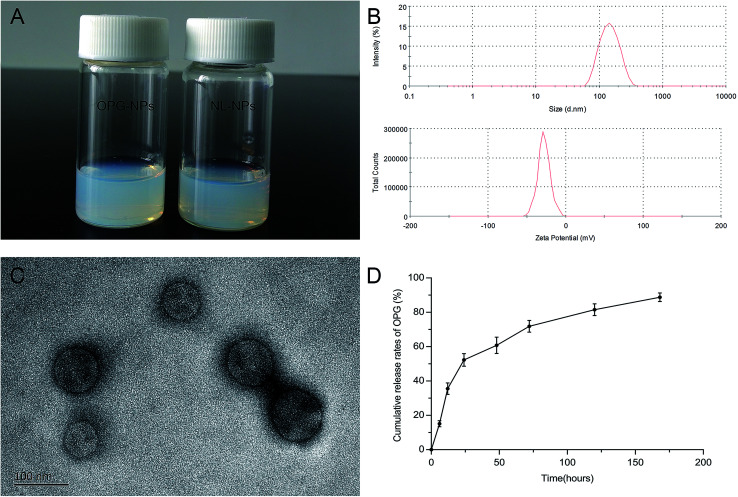
Characterization of the nanoparticles: (A) OPG-loaded nanoparticles and non-loaded nanoparticles solution, (B) particle size and zeta potential distribution of OPG-loaded nanoparticles, (C) scanning electron microscopy image of OPG-loaded nanoparticles and (D) *in vitro* release of OPG-loaded nanoparticles.

The OPG nanoparticles were fabricated *via* emulsion solvent volatilization.^20^ As shown in Fig. 2A, the OPG nanoparticle suspension prepared in this study was semi-transparent and pale blue opalescence, with no precipitation observed. Its particle size as measured by the LLS method was 133 nm, the dispersion index (PDI) was 0.131, and the zeta potential was −27.8 mV (Fig. 2B). TEM demonstrated that the prepared nanoparticles were round, regular in size, uniform in distribution, and exhibited no adhesions to each other (Fig. 2C). However, the particle size of the nanoparticles as measured by TEM was smaller than that by laser light scattering, which is due to the shrinkage of the nanoparticles after drying.^21^ Nanoparticles prepared *via* the emulsifying solvent volatilization method have a lower encapsulation efficiency for water-soluble drugs and higher encapsulation efficiency for hydrophobic drugs.^14^ In this study, water-soluble OPG and phospholipids were freeze-dried to form OPG–phospholipid complexes, which encapsulated a layer of hydrophobic phospholipids on the surface of OPG, improving the encapsulation efficiency of nanoparticles and avoiding direct contact between OPG and organic solvents.^22^ We calculated the nanoparticle encapsulation efficiency to be as high as 75% using the content of OPG wrapped in nanoparticles and the total OPG content.

### *In vitro* cumulative release of OPG

We used PBS as the release medium to detect the release of OPG for 7 days. From the release curve (Fig. 2D), it can be seen that OPG was released from the nanoparticles at a faster rate, but with no bursts during the early stage of release. The 24 hour OPG cumulative release rate reached 52%, and then the release rate remained flat. By day 7, the OPG cumulative release rate reached 89%. This experiment used phospholipid molecules to form a protective “sheath” on the surface of the OPG, slowing the movement of OPG outwards, and preventing it from being released quickly.

### Isolation and culture results of BMSCs

**Fig. 3:**
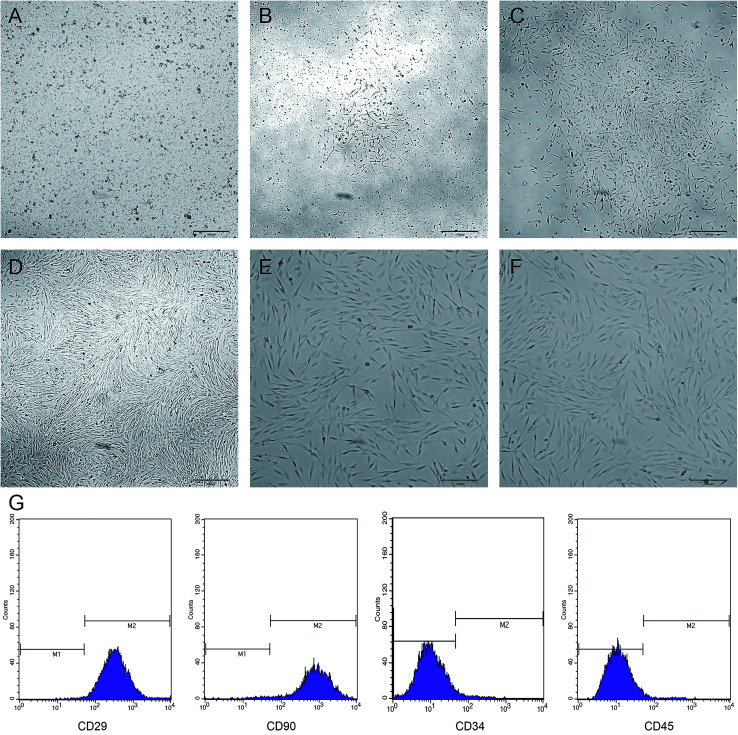
Morphological features and cell surface markers of BMSCs: (A–D) cell morphology of primary culture for 1, 3, 5, and 7 days; (E and F) cell morphology of passages 2 and 3 and (G) cell surface markers of BMSCs, respectively.

The newly inoculated cells were round and suspended in the medium (Fig. 3A). On day 3 of culture, some of the cells began to adhere to the wall and were grown in a long spindle shape (Fig. 3B). On day 5, most of the cells were already adhered to form cell colonies and gradually extended outwards (Fig. 3C). On day 7, the cells were merged by more than 70%, and then the cells were arranged in a certain direction, showing a spiral shape (Fig. 3D). After passage, the cells grew vigorously with a uniform morphology and long spindle-shaped adherent growth (Fig. 3E and F). Flow cytometry showed that the BMSCs highly expressed CD29 and CD90, but did not express or showed low expression of CD34 and CD45 (Fig. 3G).

### Effect of OPG nanoparticles on the proliferation of BMSCs

**Fig. 4:**
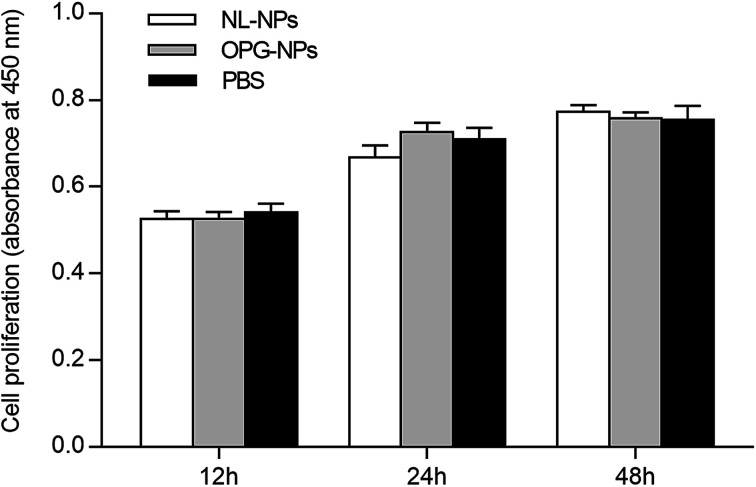
Effects of OPG-loaded nanoparticles and non-loaded OPG nanoparticles on BMSC proliferation for 12 h, 24 h and 48 h.

As shown in Fig. 4, after the addition of nanoparticles to the culture for 12 h, 24 h, and 48 h, the growth of the nanoparticles in the NL-NPs and OPG-NPs groups was not affected by the nanoparticles, showing no statistical difference between the groups (*P* > 0.05) when compared with the control group (Fig. 4). With time, the absorbance of each group also increased, indicating that the nanoparticles showed no effect on the proliferation of BMSCs and the prepared OPG nanoparticles were non-toxic to BMSCs.

### Effects of OPG nanoparticles on osteogenic differentiation of BMSCs

**Fig. 5:**
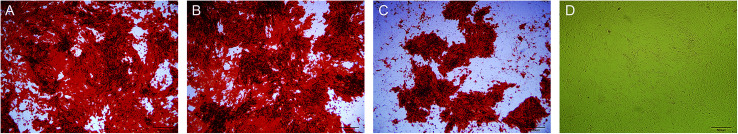
Alizarin red staining (×40): (A) cells were treated with DAG and PBS, (B) cells were treated with DAG and NL-NPs, (C) cells were treated with DAG and OPG-NPs and (D) cells were treated with complete culture medium.

The staining of the calcium nodules is shown in Fig. 5. After 21 days, the calcium deposits were stained with alizarin red to orange-red, and the DAG and NL-NPs groups showed a large number of calcium nodules clustered together, while the OPG-NPs group showed scattered calcium nodules. The number of calcium nodules was small, and no calcium nodules were found in the normal control group, indicating that OPG-NPs inhibited the differentiation of BMSCs into osteoblasts to some extent.

**Fig. 6:**
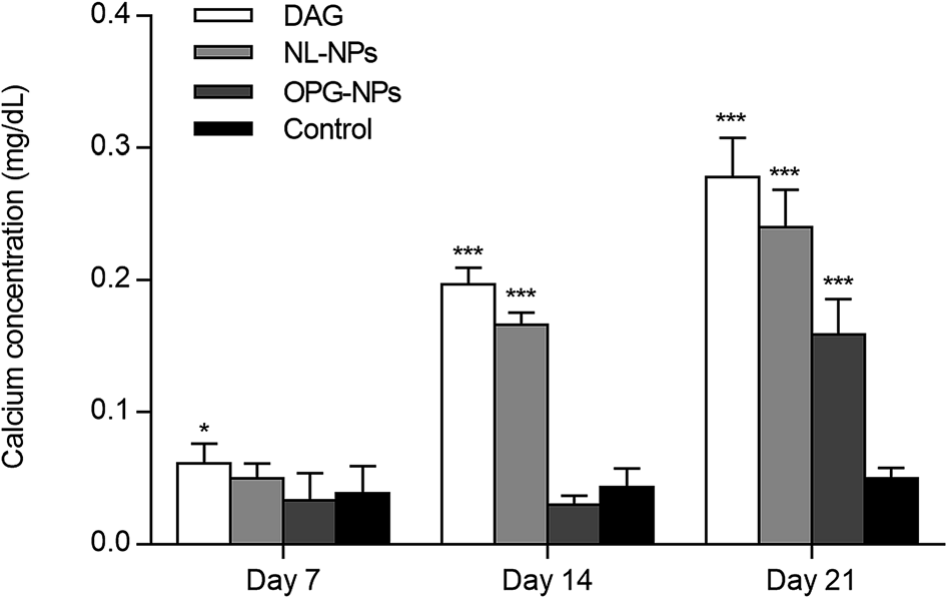
Quantitative determination of matrix calcification for 7, 14, and 21 days (*P < 0.05, ***P < 0.001).

The calcium deposition of each group was represented by the extracted calcium ion concentration (Fig. 6) on day 7 of osteoinduction. The calcium concentration in the DAG group was significantly different from that in the OPG-NPs group (*P* < 0.05). On day 14, the calcium concentration in the DAG group and the NL–OPG-NPs group increased significantly, showing a statistically significant difference compared with the OPG-NPs group (*P* < 0.001).

Up to day 21, the calcium ion concentration of each group increased continuously, with a significant increase in the DAG group, NL–OPG-NPs group and OPG-NPs group. However, the calcium ion concentration of the OPG-NPs group was still significantly lower than that of the DAG group and NL–OPG-NPs group (*P* < 0.001). There was no significant difference between the OPG-NPs group and the normal control group from 1 to 14 days (*P* > 0.05). However, on day 21, there was a significant difference in calcium ion concentration between the OPG-NPs group and normal control group (*P* < 0.001), but the OPG-NPs group showed a stronger anti-calcification effect than the DAG group and the NL–OPG-NPs group.

### Characterization of decellularized valve modified with OPG nanoparticles

**Fig. 7:**
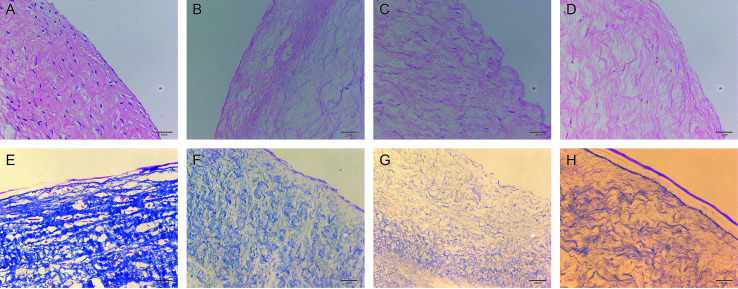
HE staining and Masson's trichrome stain results of different groups (×400): (A and E) PAV group, (B and F) DPAV group, (C and G) OPG-NPs–DPAV group and (D and H) NL-NPs–DPAV group, respectively.

The morphology of the valve tissue of each group was observed by HE and MASSON staining. In the PAV group, a large number of cells was observed, the valve structure was intact, and the fibers were in shape and continuous in a certain direction (Fig. 7A and E, respectively). No cell residue was observed in the DPAV group, and the fibers were continuously shaped in a certain direction (Fig. 7B and F, respectively). No cell residue was observed in the NPs-DPAV group, and the valve fiber structure remained intact, wavy, and the layers were clear (Fig. 7C and G, respectively). The NL-NPs–DPAV group was observed to be similar to the OPG-NPs–DPAV group (Fig. 7D and H, respectively), where the structure of the decellularized valve after nanoparticle modification remained intact.

**Fig. 8:**
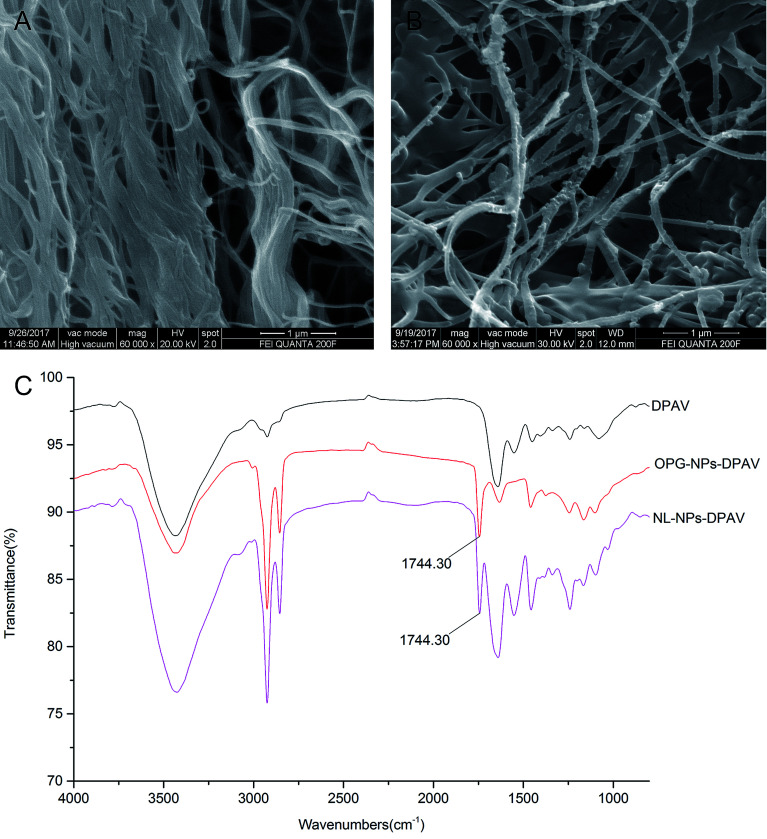
Characterization of OPG-loaded nanoparticles immobilized onto DPAV: (A and B) SEM image of DPAV and OPG-NPs–DPAV and (C) FTIR spectra of DPAV, NL-NPs–DPAV and OPG-NPs–DPAV groups.

Scanning electron microscopy demonstrated that the decellularized fibers were visible in the decellularized valve, and no cell adhesion was observed (Fig. 8A). However, the decellularized valve modified with OPG nanoparticles exhibited a layer of nanoparticles attached to the surface of the fiber, and its arrangement appeared more regular (Fig. 8B). The infrared spectroscopy results showed that most of the stretching vibration peaks of DPAV, OPG-NPs–DPAV and NL-NPs–DPAV were consistent, but OPG-NPs–DPAV and NL-NPs–DPAV showed a peak at 1744.30 cm^−1^, which is ascribed to the C=O stretching vibration peak in the nanoparticle composition, but not in DPAV (Fig. 8C). The material on the surface of the composite valve was connected with the nanoparticles, indirectly indicating that the nanoparticles were attached to the decellularized valve.

### Anti-calcification of composite valve *in vivo*

**Fig. 9:**
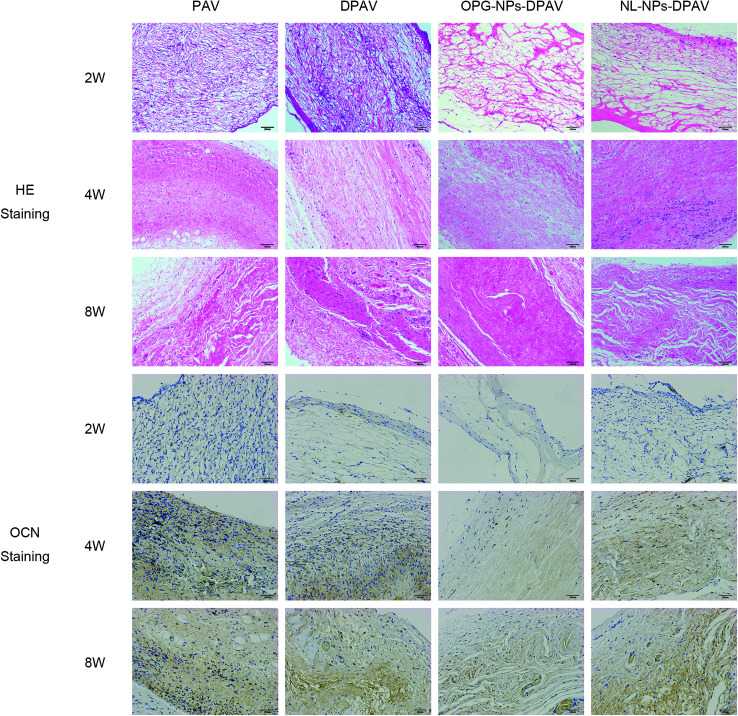
HE staining and osteocalcin immunostaining results of the different groups after subdermal implantation for 2, 4 and 8 weeks. Images were captured under 200 magnified visual field.

As shown in Fig. 9, at week 2, the valve fibers were loosely arranged in each group. There were more inflammatory cells infiltrated in the superficial layer of the PAV group and DPAV group, and small amounts of inflammatory cells infiltrated in the deep layer of the DPAV group. A small amount of inflammatory cell infiltration was observed in the superficial layer of the OPG-NPs–DPAV group and NL-NPs–DPAV group. At week 4, more inflammatory cells infiltrated in the deep layer of the DPAV group, while in the other groups, small amounts of inflammatory cells were observed in the deep layer. At week 8, the inflammatory cell infiltration was similar to that at week 4, but new capillary formation was observed in each group, with more new capillaries in the DPAV group. During the 8 weeks of implantation, the valves of each group remained in a complete shape and the fibers appeared continuous. At week 8, a small amount of degradation occurred in the valves of each group. Osteocalcin (OCN) immuno-histochemical staining (OCN was stained brown) showed low expression of OCN in the DPAV and NL-NPs–DPAV groups at week 2. At week 4, OCN expression in the OPG-NPs–DPAV group was significantly lower compared to the other groups. At week 8, a small amount of OCN expression was observed in the OPG-NPs–DPAV group, which may due to the complete release of OPG from the nanoparticles, but was still lower than the other groups. This demonstrated that the released OPG from DPAV could inhibit the expression of OCN.

OCN is a late marker of calcification in osteoblastogenesis, which appears in the later stages of skeletal bone formation.^23^ Aortic valve calcification is similar to skeletal bone formation and the process is mediated by an osteoblast-like phenotype.^24^ OPG could inhibit bone-like synthesis in the valve to prevent valve calcification. OPG strongly suppresses the levels of OCN, a protein that is specific to bone-like tissue, in an approximate proportion to suppress the valve calcification.^25^ The slow release of OPG in the composite valve prepared in this study inhibited the expression of OCN to a certain extent, indicating that the composite valve can delay the calcification of the valve.

## Discussion

This study aimed to construct a new type of tissue-engineered anti-calcification composite valve to provide a new strategy for the modification of tissue-engineered heart valves. Firstly, we prepared nanoparticles with anti-calcification factor OPG using the emulsion solvent evaporation method. The nanoparticles were uniform in size, with a encapsulation efficiency, slow *in vitro* release, non-cytotoxic to BMSCs, and delayed osteogenesis of BMSCs during osteogenesis conditions. Then, we used the unsaturated carbon–carbon double bond of the nano-particle PEG-terminal maleimide and the thiolated decellularized valve to form a Michael addition reaction to introduce the OPG-loaded nanoparticles into the decellularized valve. The decellularized valve modified by OPG nanoparticles and the fibers remained intact. Scanning electron microscopy and Fourier transform infrared spectroscopy showed that the nanoparticles were successfully bound to the decellularized valve. The *in vivo* study demonstrated that the decellularized valve modified by OPG had a certain anti-calcification ability.

Currently, all of the decellularization methods can damage the valve structure and cause potential loss of surface components, leading to a decrease in its mechanical properties. However, after biofunctionalizing DPAV with nanoparticles, the ultimate tensile strength and the fracture strength of the hybrid valve had no difference compared with PAV, indicating that the nanoparticle modification improved some of the mechanical properties of DPAV.^26^ The biocompatibility of nanomaterials decides their application prospect. In this study, the MAL–PEG–PCL modified PCL nanoparticles were prepared *via* the emulsion solvent evaporation method. PEG–PCL and PCL composed nanoparticles are nontoxic and degradable, and their degradation products are also nontoxic and environmentally friendly.^27,28^ In addition, our *in vitro* study demonstrated that the OPG-nanoparticles showed no effect on the proliferation of BMSCs. Further, our previous study also demonstrated that the decellularized valve scaffold possesses good biocompatibility.^29^

In this study, OPG and phospholipids were first freeze-dried to form OPG–phospholipid complexes, forming a protective “sheath” surrounded by phospholipid molecules around OPG, and avoiding direct contact between OPG and organic solvents. Li *et al.* prepared an insulin–phospholipid complex *via* the same method, which demonstrated good physicochemical stability in an oil solvent, allowing insulin to exert a better biological effect. In addition, the low encapsulation efficiency of hydrophilic drugs has always been one of the problems plaguing researchers.^22^ Several studies have shown that the double-emulsion solvent evaporation method improves the encapsulation efficiency of water-soluble drugs, but allows hydrophilic drugs to directly contact with organic solvents; however, it remained difficult to determine whether the activity of the drug is affected.^30,31^ The OPG–phospholipid complex prepared in this study has hydrophobic properties, which not only improved the encapsulation efficiency of hydrophilic drugs, but also protected the biological activity of OPG. In this study, osteogenic induction medium was used to induce BMSCs to differentiate into osteoblasts and simultaneously added to the medium containing OPG nanoparticles. The results showed that OPG released from the nanoparticles inhibited the calcification of BMSCs to a certain extent, but the intervention mechanism of OPG preventing the differentiation of BMSCs into osteoblasts needs further investigation.

PEG and PCL are commonly used carrier materials for the preparation of nanoparticles. Both PEG and PCL are non-toxic and have good biocompatibility, and they are approved by the FDA for human application. This study also confirmed that the nanoparticles prepared with PEG and PCL are non-cytotoxic to BMSCs. In addition, PEG is also a commonly used cross-linker for tissue-engineered heart valves.^32^ In this study, the anti-calcification biological factor OPG was introduced into the decellularized valve by using the Michael addition reaction of the unsaturated carbon–carbon double bond of the PEG-terminal maleimide of the nanoparticle carrier material and the thiolated decellularized valve, and the preparation was successfully carried out, yielding a composite valve that is resistant to calcification.

In this study, a composite valve with certain anti-calcification ability was prepared *via* tissue engineering technology and nano drug-loading technology. Subcutaneous implantation in rats showed that the composite valve began to calcify at week 8, probably because the nanoparticles were degraded in the body and completely released OPG. Therefore, the degradation of the nanoparticles and the drug loading of nanoparticles require further exploration and optimization. On the other hand, after 8 weeks of subcutaneous implantation in rats, the morphological structure of the valve was almost complete, which indicated that the composite valve has good biocompatibility. However, we did not test the blood compatibility and mechanical properties of the composite valve; thus, whether the composite valve can maintain normal function and biological performance in a complex blood flow environment still needs further research.

## Conclusions

In this study, a novel composite valve with controlled release OPG was prepared *via* tissue engineering technology and a nano drug-loading system to introduce anti-calcification biological factor OPG on a decellularized valve. *In vitro* experiments showed that the OPG nanoparticles delayed the calcification of BMSCs. BMSCs were differentiated into osteoblasts, and subcutaneous implantation experiments in rats indicated that the composite valve has anti-calcification ability to a certain extent. Further optimization and investigation into this treatment may prove beneficial for long-term anti-calcification.

## Ethical statement

All experiments were performed in compliance with the Institutional Animal Care and Use Committee (IACUC) and all experiments followed institutional guidelines. All animal procedures were performed in accordance with the Guidelines for Care and Use of Laboratory Animals of the Second Affiliated Hospital of Nanchang University. All research protocols were approved by the Institutional Animal Care and Use Committee of the Second Affiliated Hospital of Nanchang University.

## Conflicts of interest

There are no conflicts to declare.

## Supplementary Material
